# Editorial: Positive Psychology in Everyday Life

**DOI:** 10.3389/fpsyg.2022.913569

**Published:** 2022-06-01

**Authors:** Margarida Pocinho, Soraia Garcês, Daniela Popa

**Affiliations:** ^1^Department of Psychology, University of Madeira/CIERL, Funchal, Portugal; ^2^Research Center for Tourism, Sustainability and Well-Being, University of Algarve, Faro, Portugal; ^3^Department of Psychology, Education, and Teacher Training, Transilvania University of Brasov, Brasov, Romania

**Keywords:** wellbeing, quality of life, positive education, multiculturalism, tourism, special needs, sport and arts, positive work environment

## Introduction

Positive Psychology has been established as a major-based-evidence field of knowledge that aims to understand how people can improve their lives, and ultimately, flourish. Studies have been conducted since Seligman (2016) and Seligman and Csikszentmihalyi (2014) spearheaded this movement of looking for the positive aspects of life rather than focusing on the negatives.

However, although the focus of studies in the area of positive psychology is on “cultivating positive feelings, behaviors, or cognitions” (Sin and Lyubomirsky, 2009, p. 468), we wonder whether beliefs about improved wellbeing in response to positive psychology interventions do not bias the way we conduct research in this field. Recent studies show that moderators may exist in such situations from both the characteristics of the activities designed in the interventions and those of the research participants. Current research has shown that participation in activities aimed at improving wellbeing positively biases the beliefs of people in the sample (Gander et al., 2022). It is therefore more than likely that respondents will respond positively to tasks in research designs, anticipating their beneficial purpose, engage in behaviors they would not ordinarily engage in, and self-evaluate themselves as more effective than they actually are.

Despite, this growing concern, Positive Psychology has been extensively a target of research which has led, so far, to interesting results. From the benefits of positive psychology interventions in improving wellbeing and diminishing depression, anxiety or stress (Boiler et al., 2013; Carr et al., 2020), to contributing to employees' performance and productivity (Kour et al., 2019), to reducing distress in people diagnosed with clinical disorders (Chakhsii et al., 2018), or even promoting resilience and hope through specific interventions in schools settings (Platt et al., 2020). Positive psychology practices have constructive impacts on people's everyday lives such as reducing stress and anxiety, increasing resilience and promoting self-growth, wellbeing, and quality of life. This happens among different cultures, populations, contexts, and fields of knowledge, similar to the results emphasized by current meta-analyses (Koydemir et al., 2021; van Agteren et al., 2021).

Positive psychology has undeniably been a “breath of fresh air” in promoting flourishing rather than focusing only on remediation. Thus, this and other concerns most intensely discussed by each of us today are found in the themes addressed by the research on this topic. It was this diversity of results, doubts, and dispersion along with different fields of knowledge that inspire this Research Topic. The aim of this Research Topic and e-book was to explore this possible “fragmentation” of Positive Psychology and how it can lead to more dispersion or instead lead to a more unified field.

## Contributions of This Research Topic

In this Research Topic, articles were collected that highlighted the close connection between the 3 pillars of positive psychology (Seligman et al., 2009). We observed how different positive experiences, lived in various institutional settings, can contribute to the development of personality traits. Although this Research Topic appears to be an eclectic collection of research, in fact, Positive Psychology in Everyday Life reflects the multiple dimensions of the urban quotidian from an integrative perspective.

With an international editorial team of researchers specializing in Positive Psychology, this Research Topic has attracted more than 35 publications from 134 authors from around the world on different aspects of the topic. This Research Topic includes studies from 19 countries: Portugal, UK, Ireland, Italy, Spain, France, Sweden, Poland, Romania, USA, Canada, Chile, China, Japan, South Korea, Malaysia, and Brunei. Therefore, we are proud to bring together the most current theory and practice regarding positive psychology across disciplines, such as Wellbeing, Education, Tourism, Social and Organizational settings, Special needs, and Positive Psychology in multidisciplinary fields, including communication, multiculturalism, psychometrics, and cross-cultural studies.

These studies include cutting-edge ideas and research that explore multidisciplinary approaches to positive psychology in daily life and how these can contribute to reshaping the field or moving into a new “wave” of positive psychology. All studies main goals are summarized in Table 1.

**Table 1:** Summary of the contributions to this Research Topic.

**No**.	**Authors**	**Title of contribution**	**Main objectives**
1	Chen, Bao et al.	Proactive Personality and Academic Engagement: The Mediating Effects of Teacher-Student Relationships and Academic Self-Efficacy	The current study aimed to investigate the relationship between proactive personality and academic engagement.
2	Liu et al.	The Antecedents of Thriving at Work: A Meta-Analytic Review	In this study, a systematic and comprehensive meta-analysis of the relationship between thriving at work and its antecedents is conducted.
3	Farnicka et al.	Positive Psychology in Poland Between 2001 and 2020: A Review of Available Articles	Thirty intentionally selected articles were analyzed in terms of research objectives, variables, and measurement tools they described.
4	Mahmic et al.	Identifying and Shifting Disempowering Paradigms for Families of Children with Disability Through a System Informed Positive Psychology Approach	Critically examine typical approaches to disability care for families of young children, describe the importance of a systems-informed positive psychology (SIPP) approach to care, and identify the existence of two dominant paradigms, disability is a disadvantage and experts know best.
5	Zhang et al.	Belief in a Just World and Moral Personality as Mediating Roles Between Parenting Emotional Warmth and Internet Altruistic Behavior	This study explores the influence of parental emotional warmth on college students' Internet altruistic behavior, and the mediating roles of personal belief in a just world and positive moral personality traits.
6	Yurrebaso et al.	The Role of Geographical Area and Entrepreneurs' Personality	This study proposed a line of research on entrepreneurship based on the analysis of personality traits and geographical area. Its objective is to identify whether certain personality traits or sociocultural variables typical of a particular geographical area influence those who have already started an entrepreneurial activity to keep it up, in other words, to maintain their entrepreneurial intention.
7	Park	The Meaning of Musicing in the Post-traumatic Growth of Individuals With Adventitious Visual Impairment: Applying the Life History Method by Mandelbaum	This study investigated individuals with adventitious visual impairment (AVI) acquired during adulthood through a traumatic event, for an in-depth and contextual understanding of the factors and processes that led to positive changes in their post-traumatic growth.
8	Dolan and Henwood	Five Steps Towards Avoiding Narrative Traps in Decision-Making	Using the uncertainty surrounding COVID-19 as an illustration, we show how a narrative to preserve life has become dominant, and we illustrate how it has been reinforced by several behavioral biases. We argue that being able to identify and critically evaluate the impact of dominant narratives is vital to ensuring optimal decision-making.
9	Sun et al.	The Association Between the Subjective Exercise Experience of Chinese Women Participating in Square Dance and Group Cohesion: The Mediating Effect of Income	The study aimed to examine the relationship between the subjective exercise experience of participating in square dance and group cohesion and whether some variables (e.g., age, education, duration, income level, and work) play a role as mediators in the association with subjective exercise experience and group cohesion.
10	Kosugi et al.	Effectiveness of Mindfulness-Based Cognitive Therapy for Improving Subjective and Eudaimonic Well-Being in Healthy Individuals: A Randomized Controlled Trial	The present study examines the feasibility of mindfulness-based cognitive therapy and its effectiveness for improving subjective and eudaimonic wellbeing among community residents.
11	Chen, Yu et al.	The Influence of Trust on Creativity: A Review	The goal of this study is to investigate how trust influences creativity by summarizing existing findings of diverse empirical studies.
12	Battulga et al.	The Impact of Social Support and Pregnancy on Subjective Well-Being: A Systematic Review	This review aims to examine the extended association of being pregnant and SS on the SWB of pregnant women.
13	Vancappel et al.	Validation of the French Version of the Positivity Scale (P Scale)	The purpose of this study is to assess the psychometric properties of the French version of the Positivity scale (P scale), a self-report measure of positivity, which is the tendency to view and address life and experience with a positive outlook
14	Xu et al.	A Tale of Two Capitals: How Task-Oriented and Guanxi-Oriented Psychological Capitals Lead to a Sustainable Workforce in Rural China	The study aims to explore the complex relationships linking Psychological Capital and organizational commitment in a sample of public civil servants at the township level in the rural areas of northwestern China.
15	Barbry et al.	Is Football or Badminton Associated With More Positive Affect? The Links Between Affects and Sports Club Membership Among French Adolescents	The purpose of this study is to examine the differences between the affective benefits of specific sports in a group of adolescents.
16	Gilbertson et al.	Closeness to God, Spiritual Struggles, and Wellbeing in the First Year of College	The study aims to examine how closeness to God and spiritual struggles are associated with wellbeing and belonging.
17	Wu et al.	Flow as a Key Predictor of Subjective Well-Being Among Chinese University Students: A Chain Mediating Model	The study aims to investigate a conceptual model by testing flow experience of university students and their subjective wellbeing relation *via* considering the underlying mechanisms of academic self-efficacy and self-esteem within such a relationship.
18	Calheiros et al.	Youth in Residential Care: A Cross-Sectional Mediation Analysis of Youth's Perceptions of Their Social Images, Self-Representations, and Adjustment Outcomes	This study aimed to analyze the indirect associations between residential care youth's MR and their psychological adjustment (i.e., externalizing and internalizing problems) through their self-representations (SR) and test the moderating role of youth's age and residential unit size in those associations.
19	Mieres-Chacaltana et al.	Prosocialness and Happiness in Chilean Student Teachers	The aim of the study was to evaluate the relation between prosocialness and happiness in a sample of student teachers
20	Ungar and Jefferies	Becoming More Rugged and Better Resourced: The R2 Resilience Program's© Psychosocial Approach to Thriving	This paper reviews the justification for a multisystemic approach to designing resilience interventions and then explains the process of implementation of the R2 resilience program.
21	Jones and Drummond	A Summary of Current Findings on Quality of Life Domains and a Proposal for Their Inclusion in Clinical Interventions	The objective of this study is to both investigate QoL domains relevant to clinical interventions and, if applicable to identify feasible avenues for their inclusion by clinicians seeking to improve intervention efficacy.
22	Pocinho et al.	Wellbeing and Resilience in Tourism: A Systematic Literature Review During COVID-19	This research aims to analyze early positive approaches and attitudes to respond to the negative impact of COVID-19 in tourism everyday activities that have at its core wellbeing and resilience.
23	Popa et al.	Linking Positive Psychology and Intercultural Competence by Movies: Evidence From Brunei and Romania	This study investigates the wellbeing associated with learning the meanings of being different and growing in emotional resilience, flexibility, and openness to other cultures through movies.
24	Czyzowska and Ewa Gurba	Enhancing Meaning in Life and Psychological Well-Being Among a European Cohort of Young Adults via a Gratitude Intervention	The aim of the research was to explore how a gratitude intervention will affect the sense of meaning in life, psychological wellbeing, general health and perceived stress among young adults.
25	Henter and Nastasa	Parents' Emotion Management for Personal Well-Being When Challenged by Their Online Work and Their Children's Online School	The study aims to investigate the relationship between parents' emotional intelligence and their ability to manage their emotions during this COVID-19 lockdown,
26	Fratczak-Müller	Innovative Housing Policy and (Vulnerable) Residents' Quality of Life	This study focus on is the process of implementing the social housing policy and its impact on increasing the quality of life (QOL) of vulnerable people.
27	Wang et al.	The Combined Effects of Patient Activation and Relational Aspects on the Quality of Life in Atrial Fibrillation Patients	This study aims to explore the influence of patient activation (PA) and relational aspects on the quality of life (QoL) in patients with Atrial Fibrillation (AF) for developing measures to improve PA and QoL
28	Tisu and Vîrgă	Proactive Vitality Management, Work–Home Enrichment, and Performance: A Two-Wave Cross-Lagged Study on Entrepreneurs	This study provides a cross-lagged examination of the relationships between proactive vitality management, work–home enrichment, and entrepreneurial performance
29	Lim et al.	How Does Search for Meaning Lead to Presence of Meaning for Korean Army Soldiers? The Mediating Roles of Leisure Crafting and Gratitude	This study aimed to investigate the mechanisms of how individuals living in life-threatening and stressful situations obtain meaning in life, by investigating the mediating roles of leisure crafting and gratitude.
30	Wang et al.	The Effect of Place Attachment on Overseas Students' Tourism Ambassador Behavior: A Mediation Role of Life Satisfaction	This study attempts to examine the effect of place attachment and student life satisfaction on Mainland Chinese students' word-of-mouth (WOM) recommendations and their Ambassador Behavioral (AB) intention.
31	De Netto et al.	Communication, the Heart of a Relationship: Examining Capitalization, Accommodation, and Self-Construal on Relationship Satisfaction	In this study, accommodation and capitalization, were explored concurrently to disentangle the unique associations and influence on relationship satisfaction. The study also sought to understand the moderating effects of culture in terms of interdependent self-construal on the link between these two communication processes and relationship satisfaction.
32	Park and Im	Is Posttraumatic Growth Helpful in Overcoming Mental Health Disorders Due to COVID-19?: The Moderating Effect of Posttraumatic Growth in the Relationship Between COVID-19 and Psychological Health	The aim of this study was to investigate the effects of restrictions and concerns related to the coronavirus disease 2019 (COVID-19) on depression, anxiety, and committed action, and examine whether posttraumatic growth (PTG) serves as a protective factor for mental health.
33	Chen, Dou et al.	The Basic Empathy Scale in Chinese College Students: Adaptation and Psychometric Properties of a Revised Form	This study aimed to revise the Chinese version of the Basic Empathy Scale for college students.
34	Geremias et al.	Psychological Capital Profiles and Their Relationship With Internal Learning in Teams of Undergraduate Students	This study aims to analyze the relationship between psychological capital profiles and internal learning in teams.
35	Deng et al.	Job Demands and Resources and Employee Well-Being in the Chinese Nonprofit Sector	This study applies the job demands and resources (JD-R) model to investigate JD-R's relations to employee wellbeing (EWB) among Chinese foundation employees.
36	Martins et al.	Positive Development Based on the Teaching of Personal and Social Responsibility: An Intervention Program With Institutionalized Youngsters	This study aims to evaluate the effects of an intervention geared toward teaching life skills through sport to youngsters who had been committed.

The articles in this Research Topic focused on a wide variety of populations across the lifespan and included those suffering from psychological/mental problems as well as healthy children and adults. The studies were conducted in a variety of settings—schools, universities, residential care, business, tourism, and in the community—suggesting that positive psychology interventions can be done anytime and anywhere and included both short-term and long-term interventions. Beyond this, the research included in this topic has covered a wide range of methodologies, from experimental and correlational studies to systematic and comprehensive reviews. There have also been several articles that focused on participants' experiences. However, from this diversity 6 big clusters of topics also emerged.

### Positive Education

One of the education studies explores the influence of parental emotional warmth on 893 college students' altruistic behavior in the virtual environment, as well as, and the mediating roles of personal belief in a just world and positive moral personality traits (Zhang et al.). The study by Mieres-Chacaltana et al. showed a positive relationship between prosocialness and happiness in a sample of 224 students and teachers. In positive psychology, spirituality is an important variable, but often an overlooked aspect of the self that may affect college students' wellbeing and belonging. The study by Gilbertson et al. examined closeness to God and spiritual struggles as predictors of first-year college students' wellbeing. Other research investigated a conceptual model by testing flow experience and subjective wellbeing of 1.109 university students during COVID-19 considering their underlying academic self-efficacy and self-esteem (Wu et al.). In another interesting research, the relationship between psychological capital profiles and internal learning in teams was analyzed. The student profile with the highest scores in self-efficacy, optimism, hope, and resilience exhibited also the highest scores of internal learning in teams; there was no significant relationship between the profile with a positive combination between self-efficacy and hope and the profile that presents the optimism as the only positive psychological capability (Geremias et al.). Similarly, Dolan and Henwood examined how narratives provide simple rules about how we might live and what our decision-making priorities ought to be. Chen, Bao et al. showed the positive effects of Teacher-Student Relationships and Academic Self-Efficacy on Proactive Personality and Academic Engagement with 549 children. In another spectrum of positive education, parents' emotional management was highly required during the COVID-19 lockdown, juggling their job as it moved online with being a parent of a child whose school was online and that proved to be a challenge for many (Henter and Nastasa). The authors also investigated the participants' level of flourishing, as these changes impacted differently on every parent's wellbeing. The analysis of the data provided us with the opportunity to make a series of recommendations for parents' wellbeing in such a situation, as the prospect of continuing to work and learn online in the future seems very real. The need to set clear boundaries between the roles played in these settings emerged as the main objective of future therapeutic interventions based on positive psychology.

### Quality of Life of Special Needs and Vulnerable Populations

The Research Topics also gathered a cluster of articles that focused on the benefits of positive psychology approaches with vulnerable and special needs populations, where there was a strong emphasis on improving the social and emotional wellbeing and quality of life. Thus, Jones and Drummond did a summary of current findings on quality of life and wellbeing domains and a proposal for their inclusion in clinical interventions. Also, Fratczak-Müller investigated the efficacy of implementing a positive social housing program in increasing the quality of life of vulnerable people. Likewise, Calheiros et al. analyzed youth in residential care, through a cross-sectional mediation analysis of youth's perceptions of their social images, self-representations, and adjustment outcomes. The results emphasize the relevance of stimulating positive SR, by showing that they can be a protective factor for youth in residential care. In another study Wang et al. explored the influence of patient activation (PA) and relational aspects on the quality of life (QoL) in patients with Atrial Fibrillation (AF) for developing measures to improve PA and QoL. Park investigated individuals with adventitious visual impairment acquired during adulthood through a traumatic event, for an in-depth and contextual understanding of the factors and processes that led to positive changes in their life. Another research from Mahmic et al. showed the efficacy of a System of a positive psychology approach to identify and shift disempowering paradigms for families of children with disability. Finally, the article of Czyzowska and Gurba showed that strengthening the sense of meaning in life and psychological wellbeing brings benefits for mental health to a group of young adults particularly vulnerable to mental problems.

### Social and Organizational Positive Psychology

In the field of social and organizational psychology, several studies showed the benefits of the use of positive psychology “personality” in everyday work life. The role of geographical area and entrepreneurs' personality by Yurrebaso et al. proposed a line of research on entrepreneurship based on the analysis of positive personality traits. Thus, the profile of the entrepreneur, who maintains a high entrepreneurial intention, would be characterized by a high internal locus of control, a low external locus of control, high self-efficacy, proactivity, risk-taking tendencies, and personal initiative. Xu et al. indicated psychological capital as a positive variable in influencing employees' behavior and its role in maintaining a sustainable workforce in underprivileged rural China. Deng et al. investigated the factors that contribute to employee wellbeing among non-profit sectors. In the same way, Tisu and Vîrgă provided a cross-lagged examination of the relationships between proactive vitality management, work–home enrichment, and entrepreneurial performance. Liu et al. conducted a systematic and comprehensive meta-analysis of the relationship between thriving at work and its antecedents, based on a positive psychological state. Chen, Yu et al. investigated how trust influences creativity by summarizing existing findings of various empirical studies.

### Psychometric Research

The need for measures with good psychometric proprieties was also seen in the two studies that focused on psychometric research. One of the studies focused on the validation of the French Version of the Positive Scale (Vancappel et al.), a self-report measure of positivity, which is the tendency to view and address life and experience with a positive approach. The other was an adaptation of the Chinese version of the Basic Empathy Scale, with a sample of 805 college students (Chen, Dou et al.). This study showed that emotion and empathy have a significant correlation with gratitude and altruism online.

### Sports and Arts

Barbry et al. indicated the links between positive affect and sports club membership among French adolescents. Sun et al. explored physical and mental health issues in middle-aged women, demonstrating that participation in square dancing can increase women's positive subjective wellbeing and has the potential to reduce their negative emotions, which can improve their long-term health. According to Martins et al., from the standpoint of the school settings, sports participation constitutes a key strategy concerning the manifestation of positive behaviors that result from the development of personal and social responsibility. Thus, this article validated a positive development of sports intervention with institutionalized youngsters, based on the teaching of personal and social responsibility. Linking positive psychology and intercultural competence through movies was the research of Popa et al. from Brunei and Romania. Cultural consumption provides numerous benefits for individuals, especially for younger generations. Imaginary travel narratives can shape people's perceptions about other cultures and thus are useful tools for developing positive intercultural competencies.

### Improving Wellbeing

Kosugi et al. unveiled the effectiveness of mindfulness-based cognitive therapy for improving subjective and eudaimonic wellbeing in healthy individuals. The study of the processes that enrich positive relationships has been an under-researched area within positive psychology practice (De Netto et al.). The authors consider communication as the heart of a relationship, examining capitalization, accommodation, and self-construal on relationship satisfaction. Another article examined the posttraumatic growth helpful in overcoming mental health disorders due to COVID-19. It showed the moderating effect of posttraumatic growth in the relationship between COVID-19 and psychological health (Park and Im). A systematic review conducted by Battulga et al. demonstrated that subjective wellbeing (SWB) has a protective role in mental health maintenance and is prone to change during short stressful moments, such as pregnancy. An intervention resilience program focused on self-regulation and academic success, the R2 Resilience Program, was applied to clients of urban social services to workers in a long-term care facility, managers in the health care sector, staff of a Fortune 500 corporation, students in a primary to grade 12 school, and adult volunteers affiliated with an international NGO (Ungar and Jefferies). In another paper on this Research Topic it is said that many studies establish that finding meaning in life reduces stress and promotes physical and psychological wellbeing. However, extant literature focuses on meaning in life among the general population (e.g., college students or office workers). The study presented on this issue includes Korean army soldiers and aims to understand how the search for meaning leads to the presence of meaning by the mediating roles of leisure crafting and gratitude (Lim et al.). In Farnicka et al., the study showed that life satisfaction and mental wellbeing were the main subjects of interests by researchers in Poland. Finally, two papers on this Research Topic focused on the tourism field. One was a systematic literature review during COVID-19 about wellbeing and resilience in tourism, by Pocinho et al. Results showed that a positive and resilient approach to dealing with the adverse outcomes of the pandemic is a concern for stakeholders and the future of the organizations in the tourism and hospitality sector, as is tourists' wellbeing. Other research explored the effect of place attachment on overseas students' tourism ambassador behavior: a mediation role of life satisfaction, as a hotspot in positive psychology in recent years (Wang et al.).

Overall, while this strong and evidence-based body of knowledge and studies about positive psychology is a major enabler in advancing the research in this field and bringing practical tools and insights for improving peoples' everyday lives, these clusters of information provide an equally vast dispersion of research across disciplines. However, at the same time, these clusters show us that Positive Psychology can be part of everyone's daily lives. While a so-called fragmentation can be seen when we think of the multiple fields, techniques, variables and populations that were target of the different studies that compile this Research Topic, in this dispersion we can also see its importance and maybe its unification. Positive Psychology theory and practice are sought ought in every context: education, research (psychometrics), sports, arts, social settings and organizations; by a diversity of people including specific populations such as special needs and in disadvantages situations; with a common ground: improving wellbeing. In this, we can see how the dispersion of themes and interests in research can also be proof that positive psychology is possible in everyday's live, and in so, we can see its potential for a more fulfilling life in every place, culture, context and environment.

## Conclusion

In conclusion, positive psychology and its immeasurable associated variables have been the focus of a large amount of research as perhaps no other so young a science has been to date. Although fragmentation of the field in multiple directions can be seen, this does not undermine the positive benefits of this field, it highlights that today, maybe more than ever, positive psychology is needed in everyone's everyday life.

## Author Contributions

All authors listed have made a substantial, direct, and intellectual contribution to the work and approved it for publication.

## Funding

This paper was financed by National Funds provided by FCT- Foundation for Science and Technology through project UIDB/04020/2020.

## Conflict of Interest

The authors declare that the research was conducted in the absence of any commercial or financial relationships that could be construed as a potential conflict of interest.

## Publisher's Note

All claims expressed in this article are solely those of the authors and do not necessarily represent those of their affiliated organizations, or those of the publisher, the editors and the reviewers. Any product that may be evaluated in this article, or claim that may be made by its manufacturer, is not guaranteed or endorsed by the publisher.
